# Activation of Insulin Signaling by Botanical Products

**DOI:** 10.3390/ijms22084193

**Published:** 2021-04-18

**Authors:** Tovit Rosenzweig, Sanford R. Sampson

**Affiliations:** 1 Departments of Molecular Biology and Nutritional Studies, Ariel University, Ariel 4077625, Israel; 2 Department of Molecular Cell Biology, Rehovot and Faculty of Life Sciences, Weizmann Institute of Science, Bar-Ilan University, Ramat-Gan 5290002, Israel; sansampson@gmail.com

**Keywords:** phytochemicals, medicinal plants, insulin signaling, insulin receptor, PTP1B, GLUT4, type 2 diabetes

## Abstract

Type 2 diabetes (T2D) is a worldwide health problem, ranked as one of the leading causes for severe morbidity and premature mortality in modern society. Management of blood glucose is of major importance in order to limit the severe outcomes of the disease. However, despite the impressive success in the development of new antidiabetic drugs, almost no progress has been achieved with regard to the development of novel insulin-sensitizing agents. As insulin resistance is the most eminent factor in the patho-etiology of T2D, it is not surprising that an alarming number of patients still fail to meet glycemic goals. Owing to its wealth of chemical structures, the plant kingdom is considered as an inventory of compounds exerting various bioactivities, which might be used as a basis for the development of novel medications for various pathologies. Antidiabetic activity is found in over 400 plant species, and is attributable to varying mechanisms of action. Nevertheless, relatively limited evidence exists regarding phytochemicals directly activating insulin signaling, which is the focus of this review. Here, we will list plants and phytochemicals that have been found to improve insulin sensitivity by activation of the insulin signaling cascade, and will describe the active constituents and their mechanism of action.

## 1. Introduction

Type 2 diabetes (T2D) is a chronic progressive disease which develops, in part, as a result of insulin resistance. The decrease in response to insulin leads to increased insulin secretion, which is eventually followed by the failure of pancreatic beta-cells to compensate for the elevated insulin demand, resulting in hyperglycemia. Chronic hyperglycemia and hyperinsulinemia lead to significant damage in various tissues and organs and to an elevated risk for the development of severe complications. T2D was formerly considered to be mainly an adult-onset disease, but has increasingly been diagnosed in adolescent and young patients [1], perhaps as a result of increased childhood obesity. Early onset T2D further heightens the risk for diabetes-related complications because of lifetime exposure to hyperglycemia [1]. Thus, taking into account the increased prevalence and the lowered age of diagnosis, T2D is a worldwide health problem and is ranked as one of the leading causes for severe morbidity and premature mortality in modern society. The T2D pandemic has wide implications regarding quality of life and life expectancy, as well for economic and healthcare systems. In order to limit the severe outcomes of the disease, the management of blood glucose levels and other metabolic imbalances that develop in T2D is of major importance.

In addition to the benefit that may be derived from the adoption of a healthy lifestyle to prevent the development or lower the severity of T2D, numerous noninsulin-like anti-diabetic drugs have been approved for clinical use. These include drugs developed to fight hyperglycemia by interfering in the various events affecting blood glucose, such as insulin secretagogue (sulfonylureas) [2], glucagon-like peptide 1 (GLP-1) analogues, GLP-1 receptor agonists and inhibitors of dipeptidyl peptidase 4 (DPP-4), the GLP-1 inactivating enzyme. GLP-1 is a hormone secreted by entero-endocrine L cells in response to glucose absorption, and suppresses glucagon secretion from α cells while it promotes expansion of β cell mass and potentiates insulin secretion [3,4]. Metformin, the first-choice and the most frequently recommended medication for T2D treatment, improves glucose metabolism via both the activation of AMP-activated kinase (AMPK) and promotion of GLP-1 release [5]. Other mechanisms to lower blood glucose include the inhibition of intestinal glucose absorption via alpha-glucosidase inhibitors and increasing glucose elimination in the kidney through the inhibition of sodium-glucose cotransporter-2 (SGLT2). Thus, a wide panel of drugs with different mechanisms of action are available for the treatment of diabetes. Although insulin resistance is the major underlying pathology of T2D driving the progression of the disease, no drugs other than thiazolidinediones (TZDs) are specifically targeted against this disturbance. Unfortunately, although TZDs (selective PPARgamma agonists) have proven insulin-sensitizing activity, several significant safety concerns have resulted in their suspension from clinical use in most countries [6]. Thus, despite the impressive success in the development of new drugs during the last 10 years for the treatment of T2D, almost no progress has been achieved with regard to the development of novel insulin-sensitizing agents. As insulin resistance is the most eminent factor in the patho-etiology of T2D [7], it is not surprising that despite the large number of anti-diabetic drugs, an alarming number of patients still fail to meet glycemic goals [8].

Hyperglycemia is only one manifestation of the disease which is accompanied by other disturbances, including dyslipidemia, chronic inflammation and non-alcoholic fatty liver disease. These alterations are all associated with insulin resistance, either resulting from impaired insulin action, or as a result of the exaggerated levels of the hormone, which has negative consequences as well.

The plant kingdom contains more than 200,000 different secondary metabolites of immense chemical diversity. Owing to its wealth of chemical structures, the plant kingdom might be considered as an inventory of compounds exerting various bioactivities, which might be used as a basis for the development of novel medications for various pathologies [9]. Almost half of the drugs approved during the last few decades originate or are derived from natural compounds. Some antidiabetic drugs were also developed from botanical compounds, including metformin, derived from *Galega officinalis*, and SGLT2 inhibitors, agents inspired by phlorizin, a natural compound isolated from the bark of apple trees.

Antidiabetic activity is found in over 400 plant species, and is attributable to varying mechanisms of action including the stimulation of insulin secretion, inhibition of α-amylase and β-glucosidase activities and anti-oxidant function. Many other medicinal plants exert their antidiabetic activity by the alleviation of insulin resistance, either by neutralizing oxidative stress or by attenuation of inflammation. Relatively limited evidence exists regarding antidiabetic plant and phytochemicals acting directly to activate insulin signaling [10,11]. This issue is the focus of this review. Here, we will list plants and phytochemicals that have been found to improve insulin sensitivity by activation of the insulin signaling cascade. If known, the active chemical constituent and the specific mechanism of action will be described.

In vivo studies demonstrate that a large number of plants have promising effects to overcome insulin resistance. However, improved insulin sensitivity following treatment with a particular plant or phytochemical in animal studies does not allow for differentiation between direct insulin-sensitizing activity and indirect effects through the ablation of factors such as inflammation and oxidative stress that promote insulin resistance. In contrast, in vitro studies enable the potential elucidation of underlying mechanisms of action and identification of compounds (or extracts) that may directly activate insulin signaling and facilitate glucose transport. In this review, we shall focus on medicinal plants whose insulin-sensitizing properties are supported by both in vitro and in vivo data.

## 2. An Overview of the Regulation of Glucose Transport by the Activation of Insulin Signaling

The regulation of blood glucose levels is mainly accomplished by insulin, released from the β-cells of the pancreas, through its initial interaction with insulin receptors (IR) found mainly on the cell membrane of most cells. Of particular importance are those found in skeletal muscle, fat and liver cells. There have been several excellent articles in which the structure of IR and the sequelae of effects that result from IR binding to it are described in great detail [12,13,14,15,16]. Accordingly, we will present only a brief description of these events here.

IR belongs to the family of receptor tyrosine kinases and is a tetramer consisting of two identical extracellular α-subunits that bind insulin, and two identical transmembrane β-subunits that have intrinsic tyrosine kinase activity on the intracellular domain. Insulin binding induces a conformational change which allows autophosphorylation of tyrosine residues on the β subunits, and this permits access to ATP and substrate-binding sites and promotes IR kinase activity [17]. Autophosphorylation of the specific tyrosine sites in the juxtamembrane domain is important for the interaction of IR with intracellular substrates containing phosphotyrosine-binding (PTB) domains, such as insulin receptor substrate (IRS-1). In the glucose regulatory pathway, activated IRS-1 initiates the subsequent signal transduction pathway by binding and activating phosphoinositide 3-kinase (PI3K), which then activates several other kinases, most notably protein kinase B (PKB-Akt). Akt, a serine threonine kinase considered a major element in the insulin-signaling cascade, is a major downstream target of receptor protein kinases that signal via PI3K. The first step of Akt activation involves translocation to the plasma membrane where it undergoes a conformational change leading to its phosphorylation [7,18].

The primary metabolic effect of insulin is the stimulation of glucose transport in adipose tissue and skeletal and cardiac muscle. This is accomplished through the translocation of the insulin-sensitive glucose transporter 4 (GLUT4) from intracellular vesicles to the plasma membrane, a process triggered by Akt. GLUT4 is one of 13 human glucose transporter isoforms (GLUTs) with 12 membrane-spanning domains, and is highly expressed in adipose tissue and skeletal muscle. GLUT4 is mostly intracellular in vesicles in the unstimulated state and are actively redistributed to the plasma membrane in response to insulin action [7,18].

Insulin signaling is normally counteracted by well-balanced and highly coordinated activation of a set of phosphatases, that enable the inactivation and termination of the signal. IR and IRS1/2 are dephosphorylated within minutes of insulin stimulation by PTP1B, T-cell PTP (TCPT) and Src homology phosphatase 2 (SHP2), which are phospho-tyrosine specific phosphatases (PTPs) [19]. Subsequently, downstream components of the signal are dephosphorylated by lipid phosphatases (PTEN and SHIP2) and serine-threonine phosphatases (PP2A and PHLPP) that antagonize the phosphorylation of phosphatidyl-inositol-3 phosphate (PIP3) and Akt, respectively [20].

## 3. Insulin Receptor Activation by Botanical Compounds

Although the precise mechanism is still unknown, activation of the receptor by non-insulin molecules might be achieved either by their interaction with binding sites on the α-subunits of the receptor, or through the direct activation of the kinase domain of the intracellular arm of β-subunits [21,22,23,24]. In addition, some compounds might not be stand-alone IR agonists, but can sensitize the receptor, thus enabling activation of IR at sub-physiological concentrations of insulin [25].

Despite the relatively large number of natural compounds that induce transmission of insulin signaling, only few have been identified as insulinomimetic with direct activation of insulin receptor. Such non-peptidyl small molecules with insulin-like activities resist the harsh environment of the gut, and, thus, might be effective through oral administration, giving these compounds a substantial advantage over insulin therapy. Such insulin receptor activators may be expected to exert beneficial health outcomes for both type 1 and type 2 diabetic patients.

**Figure 1:**
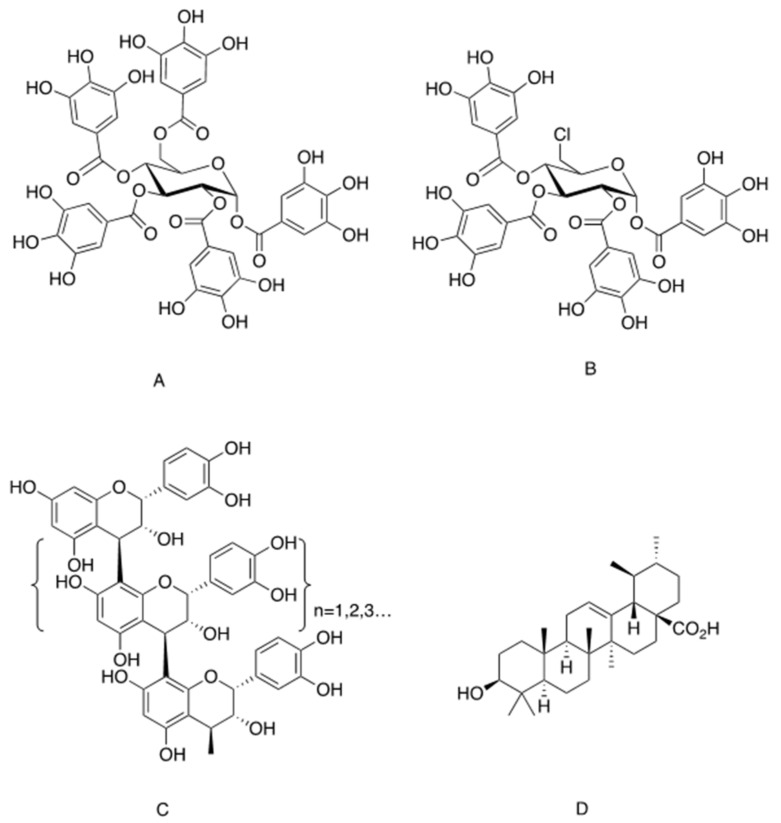
Chemical structure of phytochemicals acting directly on insulin receptor for the stimulation of insulin signaling. (**A**). 1,2,3,4,6-penta-galloyl-α-d-glucopyranose. (**B**). 6-chloro-6-deoxy-1,2,3,4-tetra-*O*-galloyl-α-D-glucopyranose. (**C**). Oligomeric procyanidins. (**D**). Ursolic acid.

One of the most investigated insulinomimetic phytochemicals is penta-galloyl-glucose (PGG), which is an ester of glucose with five gallic acid groups in the 1-, 2-, 3-, 4- and 6-positions and belongs to the large group of hydrolysable plant tannins (Figure 1A). PGG has two natural anomers, α-PGG and β-PGG, the former being more potent insulinomimetic that the later [26,27,28]. These natural gallo-tannins are found in various plants, including *Punica granatum* (pomegranate) [29], *Mangifera indica* (*M. indica*, mango) [30,31], *Lagerstroemia speciosa* (banaba) [32] and others [33]. Extracts of banaba were shown to stimulate glucose transport in adipocytes [32,34,35], and to exert antidiabetic effects in diabetic KK-Ay mice [36]. The induction of glucose transport by banaba extract was attributed to PGG rather than to other phytochemicals composing this botanical preparation [35]. Similarly, PGG isolated from *M. indica* eliminated insulin resistance and improved glucose tolerance in high fat diet (HFD)-fed mice [37,38].

PGG and its derivative, 6-chloro-6-deoxy-1,2,3,4-tetra-*O*galloyl-α-d-glucopyranose (6Cl-TGQ) (Figure 1B), were also found to alleviate glucose intolerance in several additional animal models of diabetes (ob/ob, db/db and HFD-fed mice), and to reduce blood glucose in streptozotocin-induced type 1 diabetic mice [27,39,40]. The hypoglycemic effects of PGG obtained in models of both type 1 and type 2 diabetes suggest that this molecule mimics insulin action. This idea was validated by a set of in vitro experiments on 3T3-L1 adipocytes, in which it was demonstrated that PGG and 6Cl-TGQ each binds to the α-subunit of the insulin receptor on a site different from that of insulin with higher binding affinity for the latter than the former [27,39]. The binding of these phytochemicals to IR led to the activation of PI3K, the subsequent phosphorylation of Akt and increased glucose transport in these cells.

Compounds containing galloyl groups but lacking the glucose core failed to induce glucose transport in 3T3-L1 adipocytes [28]. In addition, the presence of four galloyl groups (linked to 1, 2, 3 and 4 positions) appears to be critical for the activity of α-PGG, while all five galloyl groups are essential for the activity of the β-PGG anomer [28]. It was concluded that the glucose core is required as a scaffold for the proper spatial arrangement of galloyl groups, in order to enable the interaction of the peripheral hydroxyl groups with the binding site on the α-subunit of IR and the successive activation of the β subunits.

Possible direct activation of IR was also observed with oligomeric procyanidins, which are the oligomeric forms of epicatechin and catechin (Figure 1C). These condensed tannins can be purified from grape seeds and other botanical sources such as cocoa and pine bark [41,42]. Grape-seed procyanidins extract (GSPE), especially the fraction of trimeric procyanidins, was found to induce IR autophosphorylation in CHO-IR cells and 3T3-L1 adipocytes, which was abrogated upon treatment with IR inhibitor, HNMPA-(AM)_3_ [43]. Activation of IR and of downstream elements of the insulin signaling pathway suggests the direct activation of IR by procyanidins. However, an indirect effect, perhaps mediated via phosphatase inhibition, was not completely ruled out [43]. In addition, induction of glucose transport and activation of insulin signaling cascade were also observed with procyanidins of different levels of polymerization (dimer and tetramer), but the exact molecular target has not yet been fully clarified [44]. In vivo studies demonstrated that oligomeric procyanidins, isolated from cocoa, improved glucose and insulin sensitivity in HFD-fed mice [45]. GSPE was also found to possess hypoglycemic effects in several rodent models of diabetes. Whereas acute administration of GSPE failed to reduce blood glucose in streptozotocin rats, it did improve the hypoglycemic action of low-dose insulin [46]. In addition, by activating the PI3K-Akt pathway, GSPE appears to exert cardio-protection effects in mice [47] and may also be effective in the attenuation of various complications of the disease [48,49]. These outcomes, however, are considered to be associated with the antioxidative and anti-inflammatory properties of GSPE, rather than with its insulinomimetic effects [50].

Another potential activator of insulin signaling through a direct action on IR is ursolic acid (UA), a pentacyclic triterpenoid isolated from *Campsis grandiflora* (Figure 1D). UA is a traditional Chinese medicine [51,52]. It and other pentacyclic triterpenoids are ubiquitous in the plant kingdom, and high contents of this phytochemical were detected in apple peels (*Malus domestica*), leaves of oregano (*Origanum vulgare*), rosemary (*Rosmarinus officinalis*), sage (*Salvia officinalis*), thyme (*Thymus vulgaris*) and lavender (*Lavandula angustifolia*) [53]. UA (50 µg/mL) was reported to induce tyrosine phosphorylation of IR and subsequent phosphorylation of Akt and ERK in CHO-IR cells, indicating activation of downstream insulin-dependent metabolic and mitogenic pathways. In addition, although UA given at lower doses failed to phosphorylate IR, it intensified insulin action, leading to insulin-induced IR phosphorylation at an otherwise ineffective concentration (1 nM) [54]. It was also reported that single dose administration of UA reduced blood glucose and increased intramuscular glycogen storage in hyperglycemic rats and increased intramuscular glycogen storage [55]. On further investigation of the putative mechanism of UA action, it appeared that stimulation of glucose transport was independent of IR, as it was not abolished by HNMPA-(AM)_3_ (an insulin receptor tyrosine kinase inhibitor). In contrast, inhibition of PI3K by wortmannin successfully blocked UA-induced glucose transport [55]. It was suggested that concentrations of UA above 50 ug/mL are required to induce the activation of IR [54], whereas lower doses activate the insulin signaling cascade by the regulation of downstream components of the pathway, such as phosphatase inhibition, as will be described below [56]. Thus, UA might be considered as insulinomimetic only when given at very high doses, while most of its antidiabetic properties should be attributed to the insulin-sensitizing function of this molecule.

In summary, only a few insulinomimetic phytochemicals have been identified so far, and most evidence supports PGG as a true agonist of the receptor. However, in order to bring these molecules into clinical use, concerns related to cell toxicity, low receptor specificity and general inhibition of protein tyrosine phosphatase 1B (PTP1B) must be addressed. Specifically, because of the structural similarity of IR and other receptors of the tyrosine kinase family, mainly insulin-like growth factor 1 (IGF1) receptor, the selectivity of any insulin receptor agonist for IR must be demonstrated.

## 4. Inhibition of Phosphatases for the Activation of Insulin Signaling

An increase in IR phosphorylation does not inevitably indicate a direct activation of the receptor. Indeed, most compounds that induce tyrosine phosphorylation of the β-subunits of the receptor act via the inhibition of certain PTPs, which de-phosphorylate tyrosyl residues on IR and play a role as negative regulators of insulin signaling. Rodent and human studies demonstrated that PTPs have a major role in the attenuation of IR phosphorylation observed in diabetic patients. Elevated PTP levels have been observed in diabetic patients, while the inhibition of these negative regulators enhances phosphorylation of IR and IRS-1, and activated both PI3K-Akt and ERK pathways in CHO-IR and L6 cells [57]. It was further demonstrated that inhibition of PTP1B in the resting state is responsible for the insulinomimetic effects of the inhibitors. Inhibition of PTP1B during receptor activation leads to insulin sensitization as a result of a reduced rate of receptor dephosphorylation and prolonged activation of the signal [57]. Deletion of PTP1B potentiated insulin action and protected mice from diet-induced obesity and insulin resistance, suggesting that PTP1B is a negative regulator of some additional proteins such as the JAK2, a non-receptor tyrosine kinase activated by the leptin receptor [58,59,60]. Thus, ablation of PTP1B activity enhances both insulin and leptin signaling, leading to the mutual alleviation of obesity and insulin resistance [59]. Accordingly, in view of the search for novel drugs that improve insulin sensitivity, PTP1B is a promising target [61,62].

Unfortunately, the search for PTP1B inhibitors that are compatible for drug development is highly challenging. Although a large number of PTP1B inhibitors have been identified so far, only a negligible number of these compounds are in the pipeline to the development and approval of drugs [63,64]. The hurdles in the development of PTP1B inhibitor for therapeutic use are related to the structural complexity of PTPs and the high conservation of the catalytic active site (PTP loop) among members of the PTP family, properties that challenge the necessity for high selectivity of PTP1B inhibitors. Another obstacle in the development of PTP1B inhibitor for clinical use is their low access to intracellular destinations. This is because of the negative charge of compounds, targeting the PTP1B active site as competitive inhibitors of the phosphotyrosine substrate [65]. In fact, it was suggested that inhibitors acting via allosteric docking might elicit higher selectivity than inhibitors of the catalytic site [66]. In addition to the catalytic site, PTP1B contains several domains that promote substrate recognition, binding and catalysis [67]. The WPD-loop, a domain containing the conserved tryptophan–proline–aspartate (WPD) motif, is considered to be the regulatory switch of the catalytic site. It is a flexible domain that undergoes dynamic conformational changes of either an open, enzymatically inactive, or a closed, active state in which it moves toward the catalytic site and accelerates its action [67]. Other regulatory domains are in the Q-loop, which is the less conserved region and contributes to the activity of the Cysteine residue, which is a major component of the catalytic site and also maintains WPD in its open structure. Similarly, the E-loop is required for stabilizing the WPD loop in its closed conformation upon substrate binding [67]. As the regulatory loops are less polar and much less conserved than the active site, PTP1B inhibitors that target these domains might be promising in view of selectivity and bioavailability [63].

### 4.1. PTP1B Inhibition by Botanical Compounds

As mentioned above, several botanical compounds enhanced glucose transport when given at high doses, whereas much lower amounts had prominent effects to induce glucose transport when given with insulin, suggesting that these compounds sensitize cells to insulin action [68]. Although the mechanism underlying such phenomena might be related to synergism or cooperativity in binding capabilities to the receptor, the most reasonable explanation is that these compounds inhibit IR dephosphorylation, thus enabling amplification of insulin’s effect.

High-throughput screening of a library of traditional medicine compounds enabled the identification of ursolic acid (UA), which is a member of the ursan family of pentacyclic triterpenoids, as a competitive inhibitor of PTP1B, TCPTP and SHP2 with IC_50_ values of 3.08, 3.33 and 2.73 µM, respectively. All these phosphatases are non-receptor PTPs, while UA did not affect the catalytic function of receptor-type PTPs. UA and UA0713, which is a high potent novel derivative, induced IR phosphorylation in CHO-IR cells and glucose transport in L6 myotubes, effects that were significantly enhanced in the presence of insulin, demonstrating the potential application of these compounds in the treatment of insulin resistance [56].

**Figure 2:**
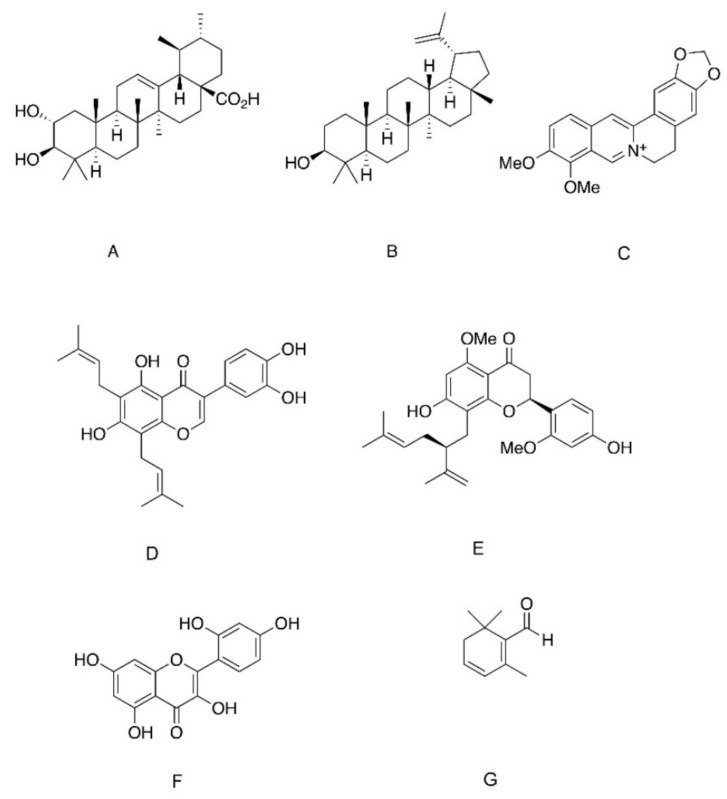
Chemical structure of phytochemicals inhibiting PTP1B activity. (**A**). Corosolic acid. (**B**). Lupeol. (**C**). Berberine. (**D**). 6,8-diprenylorobol. (**E**). (2S)-2′-methoxykurarinone. (**F**). Morin. (**G**). Safranal.

Corosolic acid (Figure 2A) is another member of the ursan family of triterpenoids that exerts PTP1B inhibitory properties [69]. Corosolic acid (CA) is a component of several medicinal plants, including *Lagerstroemia speciose, Symplocos paniculate* and *Eriobotrya japonica*, which were all found to have hypoglycemic effects [69,70,71]. When administrated to CHO-IR cells, CA had minor stimulatory effects on basal phosphorylation of IR, while significantly enhancing insulin-induced IR phosphorylation. In addition, as demonstrated for UA [56], CA inhibited PTP1B, TCPTP and SHP2 with IC_50_ values of 5.49, 11.31 and 10.50 µM, respectively, while receptor PTPs were not affected [72].

In addition to ursolic and corosolic acid, which belong to the ursan family of the triterpenes, the plant-derived lupane family of pentacyclic triterpenoids was also found to inhibit PTP1B [53,63]. Lupane triterpenoids are composed of four six-rings and one five-ring, in contrast to the members of ursan family, That are triterpenoids of five six-rings (Figure 2B). Lupane triterpenoids are isolated from *Lophopetalum wallichii*, *Bombax ceiba* and *Sorbus commixta* [73,74,75]. Members of this family include lupeol, lupenone, betulin and betulinic acid, and all were found to be non-competitive PTP inhibitors, indicating that these compounds act via allosteric inhibition of the enzyme and do not target the active site [63,73]. This assumption was supported by molecular docking and molecular dynamic simulations suggesting the role of α7 loop of PTP1B in the formation of hydrophobic interaction with lupane triterpenoids. Binding of these inhibitors to the α7 loop maintained WPD in the open position, thus inhibiting the catalytic function of PTP1B. In accordance with this allosteric mode of inhibition, lupane triterpenes, especially lupenol and betulinic acid, have high potency in the inhibition of PTP1B over TCPTP, indicating the high selectivity of the compounds [63]. In line with its inhibitory effect on PTP1B, naturally occurring lupeol and its synthetic analogues enhanced basal and insulin-induced glucose transport in L6 myotubes. This was accompanied by the activation of IRS1-PI3K-Akt pathway and the translocation of GLUT4 to the plasma membrane [75].

In addition to the triterpenoids, berberine, an alkaloid purified from the stem, roots and rhizomes of several plants including *Berberis vulgaris*, *Hydrastis canadensis* and *Cortidis rhizome*, was found to be an effective inhibitor of PTP1B (Figure 2C). Although it was found to be a less potent PTP1B inhibitor than UA (IC_50_ of berberine and UA: 16.43 and 3.91 µM, respectively) [76], berberine successfully activated insulin signaling in L6 and 3T3-L1 cells and improved glucose tolerance and insulin sensitivity in diabetic db/db mice and diabetic rats [77,78]. Based on molecular docking studies, it was predicted that berberine tightly binds to the WPD loop of PTP1B [76]. Berberine binding is expected to reduce the flexibility of this domain, maintaining its structure in the open state; thus, its mobility toward the catalytic PTP domain is impaired and phosphatase activity is diminished. In addition to this predicted allosteric regulation, binding of berberine to enzyme-substrate complex was also suggested, through binding to the pocket site [76]. These in vitro data are supported by results of clinical trials, demonstrating that berberine (1–1.5 gr/day), given for three months to type 2 diabetic patients, reduced fasting and postprandial glucose levels as well as HgA1C [79,80,81].

Several flavonoids compounds were also identified as allosteric inhibitors of PTP1B. These include 6,8-diprenylorobol (Figure 2D), a flavonoid of the plant *Flemingia philippinensis* [82], 2′-methoxykurarinone (Figure 2E), a flavonoid of *Sophora flavescens* [83], and morin (Figure 2F), which is abundant primarily in the family of *Moraceae* [84]. All these compounds displayed non-competitive PTP1B inhibition, with Ki at the micromolar range. Computational investigation modeled the molecular interaction of these compounds with PTP1B, and demonstrated van der Waals interaction with α3 and α7 regulatory domains, the more negative the van der Waals, the stronger the binding affinity and more efficient the immobilization of the WPD loop [85].

Presumably mediated by PTP1B inhibition, Morin was shown to possess insulin-mimetic properties in HepG2 hepatocytes, leading to enhanced glycogenesis and reduced glucose production, with a selective phosphorylation of IR, while EGFR and PDGFR were not affected [86]. Unfortunately, there are no sufficient data demonstrating the hypoglycemic effects of morin in rodent models of insulin resistance. The hypoglycemic properties of 6,8-diprenylorobol and 2′-methoxykurarinone require further investigation to confirm their potential use as anti-diabetic compounds.

Safranal, a component of saffron (*Crocus sativus*), is a β carotene, which was identified as a PTP1B inhibitor (Figure 2G). It was shown that safranal induces ligand-independent activation of the insulin signaling cascade and stimulation of glucose transport in C2C12 myotubes. In addition, a moderate improvement in glucose tolerance was observed in diabetic KK-Ay mice following two-weeks of safranal administration. However, the selectivity of this compound toward PTP1B was not addressed; this is an important issue, especially in view of the data demonstrating that safranal targets the cysteine residue of the catalytic site [87].

In summary, although inhibition of PTP1B is a promising target for the development of antidiabetic drugs, identification of a potent inhibitor with high selectivity and bioavailability has been extremely challenging. Combining in silico, in vitro and in vivo approaches might be an efficient path to optimize the search for botanical compounds with this anticipated activity.

### 4.2. Inhibition of Other Phosphatases by Botanical Products

Whereas large number of composites were identified as inhibitors of PTPs, much fewer compounds targeting serine-threonine phosphatases such as PP2A have been identified or synthesized. Inhibition of PP2A by a botanical preparation was only described for *Urtica dioica* (*U. dioica*, stinging nettle). *U. dioica* extract enhanced insulin-dependent phosphorylation of Akt and heightened insulin-induced glucose transport and glycogen synthesis in FFA-treated adipocytes and myotubes [88,89]. This was accompanied by attenuation of FFA-induced hyperactivation of PP2A in C2C12 myotubes despite the accumulation of ceramides, which are known activators of this phosphatase [89]. Thus, *U. dioica* inhibited PP2A via a ceramide-independent mechanism. In support of the effects of *U. dioica* extract on activation of insulin signaling, leaf extract of *U. dioica* had hypoglycemic effects in normoglycemic rats [90] and attenuated glucose intolerance and improved insulin sensitivity in HFD-fed mice [89,91]. The active components mediating the anti-diabetic effects of this botanical preparation have not yet been determined.

## 5. GLUT4 Translocation

Glucose transport is one of the final steps in the insulin signaling cascade. Stimulation of glucose transport requires the translocation of GLUT4 from intracellular stores to the plasma membrane (PM). This event is mediated by Akt, which phosphorylates AS160 (TBC1D1), a GTPase activating protein. Under basal conditions, AS160 maintains Rab proteins in an inactive GDP-bound state; however, upon phosphorylation, AS160 dissociates from Rab proteins, enabling their switch into an active GTPases [92,93].

In order to enable the induction of glucose transport, a parallel signaling cascade should be activated. The Cbl-CAP-CrkII-C3G-TC10 pathway is activated by insulin, independent of the PI3K-Akt pathway. IR phosphorylation recruits APS, which in turn undergoes phosphorylation and enables the binding of Cbl/CAP complex and phosphorylation of Cbl. This complex migrates to lipid raft subdomains of the PM and triggers the recruitment of CrkII/C3G. As a result, C3G, a guanyl nucleotide exchange factor (GEF), activates TC10, a member of the Rho family of small GTPases [93].

These two insulin-dependent pathways (PI3K-Akt-AS160-Rab and Cbl-CAP-CrkII-C3G-TC10 pathways) induce an GDP-GTP switch and activate small G proteins, an important step in the trafficking of GLUT4 storage vesicles (GSV) to the PM [94]. Transport of GSV into the close vicinity with the PM is followed by the docking and fusion of the vesicles, with the support of SNARE complexes of proteins [93,95].

Although insulin is the major factor inducing glucose transport, several other signaling pathways converge on the activation of small G-proteins to the induction of GLUT4 translocation. Among these alternatives signaling pathways, the AMPK-dependent pathway seems to play an important role in the stimulation of non-insulin dependent glucose transport. AMPK stimulates the expression of Cap and the phosphorylation of Cbl, leading to activation of the Cbl-Cap pathway for the induction of GLUT4 transport [96]. In addition, by phosphorylating AS160 on Ser711, AMPK increases insulin sensitivity upon physical activity [97,98].

In addition to the translocation of existing transporters, an increase in the pool of GLUT4 proteins promotes elevation in glucose uptake upon insulin stimulation. Transcription of the SLC2A4 gene, encoding for GLUT4, is repressed in type 2 diabetic individuals [99,100] and is enhanced by insulin in skeletal muscle [101], indicating that improvement of insulin sensitivity increases not only Glut4 translocation, but also the level of available transporters. In addition to insulin, AMPK also increases GLUT4 levels through the recruitment of transcriptional activators such as myocyte enhancer factor 2 (MEF2) and GLUT4 enhancer factor (GEF) [102].

### Botanical Products Affecting GUT4 Expression, Translocation and Functionality

**Figure 3:**
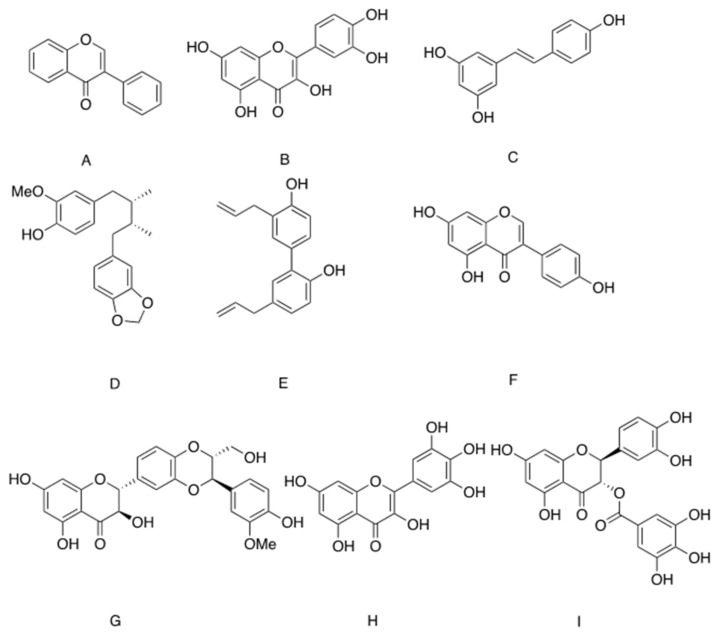
Chemical structure of phytochemicals stimulating GLUT4 expression (isoflavin (**A**), quercetin (**B**,**C**) resveratrol) or inhibiting its activity macelignan (**D**), honokiol (**E**), genistein (**F**) silybin (**G**), myricetin (**H**,**I**) catechin-gallate.

A large number of botanical products have been found to exert antidiabetic properties through the stimulation of GLUT4 translocation and the induction of glucose transport [103]. However, while many of these bioactive phytochemicals act on upstream components of the signals, either through the activation of PI3K-Akt pathway or via the activation of AMPK [103], some botanical preparations stimulate glucose transport via the induction of Slc2A4 transcription, leading to acceleration of glucose transport upon insulin induction. An isoflavone isolated from *Pterocarpus marsupium* (Figure 3A) stimulated glucose transport in L6 myotubes via a mechanism involving the transcription and translation of Scl2A4/Glut4 [104]. Another phytochemical affecting GLUT4 levels is quercetin, one of the most abundant bioflavonoids (Figure 3B). GLUT4 levels were restored and glucose transport was normalized by quercetin in palmitate-treated insulin resistant L6 myotubes. This effect was achieved by the inhibition of nuclear accumulation of nuclear factor-kappa B (NFκB) and its binding to Slc2A4 promoter [105]. Resveratrol (Figure 3C), a flavonoid molecule found at high levels in the skin of grapes (*Vitis vinifera*) and cranberry (*Vaccinium macrocarpon*) [106], reversed the reduction in Slc2A4 levels observed in T2D mice by preventing tri-methylation at lysine 9 of histone 3 (H3K9me3) in the enhancer segment of Slc2a4. This epigenetic modification enables MEF2A/D binding and upregulation of Slc2A4 transcription [107]. However, contradictory data were observed when resveratrol was investigated in vitro, in studies demonstrating that resveratrol stimulated glucose transport in C2C12 myotubes through the induction of GLUT4 translocation to the PM with no effect on GLUT4 levels [108]. All these flavonoid compounds are members of the phytoestrogen family, which might indicate the role of estrogen receptors in the regulation of glucose transport, at least in some cases. In line with this, estrogen replacement as well as the administration of phytoestrogen-rich diet or resveratrol increased GLUT4 expression and ameliorated HFD-induced glucose intolerance and insulin resistance in ovariectomized rats [108,109]. It was suggested that by binding to estrogen receptor 1 (ESR1), phytoestrogens induce an ESR1-mediated enhancer effect for the stimulation of Slc2a4 expression and improvement in glucose tolerance [110,111]. However, because activation of ESR2 repressed GLUT4 expression, the issue of phytochemical effect on glycemic control is highly complicated and depends on the tissue-specific relative expression of ESR1/ESR2 [112].

GLUT4 expression is also stimulated by ligand-bound peroxisome proliferator-activated receptors γ (PPARγ), which is a ligand-activated transcription factor that induces adipogenesis, improves insulin sensitivity and prevents inflammation. Synthetic PPARγ agonists of the TZD family elevate GLUT4 expression and glucose transport in adipocytes and ameliorate glucose intolerance in obese, diabetic mice [113,114]. The use of the luciferase reporter model enables the identification of a large number of phytochemicals as partial or full agonists of PPARγ. By attachment to the ligand-binding domain (LBD) of PPARγ, these phytochemicals mimic at least part of PPARγ functions. The large size of the binding site cavity enables the binding of structurally distinct natural compounds, which belong to seven different structural classes. This issue was nicely reviewed by Wang et al. [115].

In accordance with this, it was reported that macelignan (Figure 3D), a lignan isolated from *Myristica fragrans Houtt* (mace), is a PPARγ ligand which increases the transcription of Slc2A4 among other PPARγ target genes in 3T3-L1 adipocytes and in db/db mice [116]. Glucose intolerance and insulin sensitivity were both reduced in db/db mice treated with macelignan [116]. Honokiol (Figure 3E) is another lignan isolated from several species of *Magnolia* (Figure 3), and is a partial PPARγ agonist, as well as an agonist of retinoid X receptor (RXR), which is the dimer activation partner of PPARγ [117,118]. Through its binding to the LBD, honokiol stimulates the expression of some, but not all, PPARγ target genes. Thus, although honokiol increases glucose transport in 3T3-L1 adipocytes and exerts antidiabetic properties in KK-Ay mice, it does not stimulate adipogenesis [118].

Aside from the induction of Slc2A4 transcription and the elevation of GLUT4 levels, we did not find documentation for a botanical preparation or phytochemical that activates glucose transport through a direct interaction with GLUT4 or with the machinery responsible for GLUT4 translocation. On the other hand, while some members of the flavonoid family were reported to stimulate the expression of Slc2A4 gene and glucose transport, other phytochemicals of the same family were identified as inhibitors of glucose transport mediated by GLUT1 and/or GLUT4 [119,120,121]. These botanicals have been investigated for their potential function as inhibitors of the growth of cancer cells, which rely on glucose as a major energy source [122]. Genistein (Figure 3F), a naturally occurring isoflavone phytoestrogen found in soybean, lupin and several other plants, inhibited insulin-dependent glucose transport in adipocytes. This effect was independent of IR and Akt phosphorylation [123,124,125] and attenuated insulin sensitivity in normal mice [125]. Interestingly, by activating AMPK and negating inflammation, genistein reversed glucose intolerance induced by inflammatory stimuli in mice [125]. Silybin (Figure 3G), the major flavonoid of *Silybum marianum* (milk thistle) and its derivative dehydrosilybin, also inhibited glucose transport, while not affecting the transmission of insulin signaling and translocation of GLUT4 to the PM. These data suggest a direct inhibition of GLUT4, as was confirmed by kinetic analysis demonstrating that silybin and dehydrosilybin are competitive inhibitors of GLUT4, with Ki = 60 and 116 µM, respectively [126]. Similarly, quercetin, myricetin and catechin-gallate, all flavonoids [127,128] (Figure 3B,H,I, respectively), competitively inhibited insulin-induced glucose transport in adipocytes. A direct interaction of quercetin and catechin-gallate with GLUT4 was revealed by applying a computer simulation [120,124]. These observations appear to contradict other data demonstrating beneficial effects of quercetin on glucose transport in insulin resistant myotubes [105]. It seems, however, that while quercetin has a direct inhibitory effect on GLUT4 [120] by exerting an anti-inflammatory function, this molecule prevents the repressing effect of NFκB on GLUT4 expression. Thus, by altering glucose binding to the transporter, certain polyphenols might inhibit glucose transport, opposing the anti-diabetic properties of other phytochemicals. One may assume that a given plant extract might be composed of several distinct components with contrasting activities. The opposing activities manifested by various phytochemicals might explain why the observed effect of a crude plant extract is usually much lower than expected, compared to the anti-diabetic activity of the isolated compound of the same concentration [129].

## 6. Summary and Conclusions

**Figure 4:**
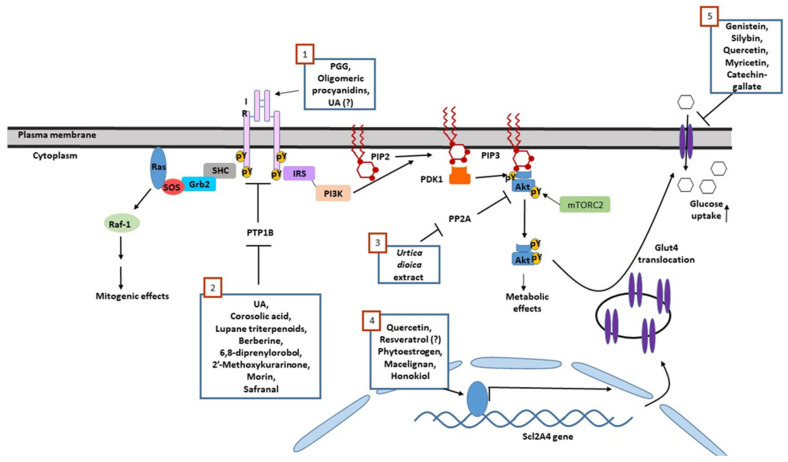
A schematic representation of insulin signaling and phytochemicals regulating its transmission. Phytochemicals stimulate insulin signaling by direct activation of insulin receptor (**1**), inhibition of PTP1B (**2**), inhibition of PP2A (**3**) and induction of Slc2A4 expression (**4**). On the other hand, certain phytochemicals might antagonize insulin signaling through the inhibition of GLUT4-mediated transport (**5**).

Although antidiabetic properties have been attributed to a large number of plants, we found that only a limited number of phytochemicals have been reported to act directly on components of the insulin signaling cascade. Activation of the insulin receptor, inhibition of PTP1B, and stimulation of GLUT4 expression are the major mechanisms mediating the effects of these insulinomimetic botanicals, leading to the induction of glucose transport and the improvement of glucose tolerance in diabetic mice. On the other hand, some phytochemicals might antagonize insulin function as a result of GLUT4 inhibition (summarized in Figure 4 and Table 1).

Phytochemicals with therapeutic functions might be consumed either as an isolated compound, or as a component of a mixture, composed of a large number of various molecules. In some cases, antidiabetic effects of a phytochemical within a botanical mixture, might be lower than the activity of the isolated one, as a result of an antagonistic inhibitory activity of other components of the same plant. In contrast, in some other botanical remedy, certain components of the preparation, might act in an additive or a synergistic pattern with activators of insulin signaling [130]. Such outcomes might be achieved by phytochemicals exerting anti-inflammatory or anti-oxidative effects, intensifying further the beneficial activity of insulinomimetics. Thus, in order to optimize the antidiabetic function of certain phytochemicals, a thorough investigation of each plant preparation should be performed, and the potency of the whole mixture should be compared with that of the isolated compound.

Despite the significant progress made in the field of phytomedicine during the last few decades, the precise composition and mechanisms of action of a large number of anti-diabetic medicinal plants are still unknown. The identification of the chemical composition of botanical preparations and isolation of active compounds are necessary for the optimization of efficacy and to ensure safety. Unravelling the mechanisms of action of identified bioactive phytochemicals is an additional important step in phytochemical research and must be comprehensively investigated in order to identify novel activators of insulin signaling and to promote effective and safe application of botanical products into the clinic.

## Figures and Tables

**Table 1:** A summary of phytochemicals regulating the transmission of insulin signalling. See the text for details.

Compound (Chemical Family)	Natural Origin	Mechanism of Action
Insulin Receptor Activators		
1,2,3,4,6-penta-galloyl-α-d-glucopyranose (hydrolysable tannins)	*Punica granatum*, *Mangifera indica*, *Lagerstroemia speciosa*	Binds to the α-subunit of the IR and induces its autophosphorylation
Oligomeric procyanidins (condensed tannins)	Cocoa, pine bark, grape seeds	Induce IR autophosphorylation
Ursolic acid (triterpenoids)	*Campsis grandiflora*, *Malus domestica, Origanum vulgare, Rosmarinus officinalis*, *Salvia officinalis*, *Thymus vulgaris*, *Lavandula angustifolia*	Insulinomimetic only when given at high doses (50 µg/mL). Intensified insulin action via phosphatase inhibition when given at lower doses
**Phosphatase Inhibitors**		
Ursolic acid (triterpenoids)	As depicted above	Competitive inhibitors of PTP1B, TCPTP and SHP2 Enhance insulin-induced IR phosphorylation
Corosolic acid (triterpenoids)	*Lagerstroemia speciose, Symplocos paniculate and Eriobotrya japonica*	Allosteric inhibitors. Bind to WPD loop of PTP1B, leading to a non-competitive PTP inhibition
Lupane (triterpenoids)	*Lophopetalum wallichii*, *Bombax ceiba, Sorbus commixta*	
Berberine (alkaloids)	*Berberis vulgaris*, *Hydrastis canadensis, Cortidis rhizome*	
6,8-diprenylorobol (flavonoids)	*Flemingia philippinensis*	
2′-Methoxykurarinone (flavonoids)	*Sophora flavescens*	
Morin (flavonoids)	*Moraceae*	
Safranal (β carotene)	*Crocus sativus*	PTP1B inhibitor. Targets the cysteine residue of the catalytic site
**Inducers of GLUT4 Expression**		
Isoflavin (isoflavones)	*Pterocarpus marsupium*	Stimulates the transcription of Scl2A4/Glut4
Quercetin (flavonoids)		
Resveratrol (flavonoids)	*Vitis vinifera* *Vaccinium macrocarpon*	
Macelignan (lignans)	*Myristica fragrans Houtt*	PPAR agonist
Honokiol (lignans)	*Magnolia*	Partial PPAR agonist
**Inhibitors of GLUT4-Mediated Transport**		
Genistein (isoflavones)	soybean, *lupinus*	Competitive inhibitors of GLUT4, inhibit insulin-dependent glucose transport. However, metabolic benefits might be achieved through anti-inflammatory effects.
Silybin (flavonoids)	*Silybum marianum*	
Quercetin (flavonoids)	Widely distributed in plants	
Myricetin (flavonoids)	* Comptonia peregrina * * Morella cerifera * * Polygonum bellardii *	
Catechin-gallate (flavonoids)	*Camellia sinensis*	

## Abbreviations

AMPK	AMP-activates kinase
APS	Adapter protein with a PH and SH2 domain
AS160	Akt substrate of 160 kDa
CA	Corosolic acid
ERK	Extracellular-signal-regulated kinase
ESR	Estrogen receptor
GEF	Guanine nucleotide exchange factor
GLP-1	glucagon-like peptide 1
GLUT4	Glucose transporter 4
GSPE	Grape-seed procyanidins extract
GSV	GLUT4 storage vesicles
IR	Insulin receptor
IRS-1	Insulin receptor substrate 1
LBD	Ligand binding domain
MEF2	Myocyte enhancer factor 2
PGG	Penta-galloyl-glucose
PI3K	Phosphatidyl insoitol-3-kinase
PKB	Protein kinase B
PM	Plasma membrane
PP2A	Protein phosphatase 2A
PPAR**γ**	Peroxisome proliferator-activated receptors γ
PTP1B	Protein tyrosine phosphatase 1B
HFD	High fat diet
RXR	Retinoid X receptor
SGLT2	Sodium-Glucose transporter 2
SHP2	SH2 containing protein tyrosine phosphatase-2
TCPTP	T-cell protein tyrosine phosphatase
T2D	Type 2 diabetes
TZD	Thiazolidinediones
UA	Ursolic acid
